# Long-term safety, discontinuation and mortality in an Italian cohort with advanced Parkinson’s disease on levodopa/carbidopa intestinal gel infusion

**DOI:** 10.1007/s00415-022-11269-7

**Published:** 2022-07-25

**Authors:** Federica Garrì, Francesco Paolo Russo, Tommaso Carrer, Luca Weis, Francesca Pistonesi, Michele Mainardi, Michele Sandre, Edoardo Savarino, Fabio Farinati, Francesca Del Sorbo, Paola Soliveri, Daniela Calandrella, Roberta Biundo, Miryam Carecchio, Anna Lena Zecchinelli, Gianni Pezzoli, Angelo Antonini

**Affiliations:** 1 Parkinson and Movement Disorders Unit, Study Center on Neurodegeneration (CESNE), Department of Neuroscience, University of Padua, Padova, Italy; 2 Parkinson Institute, ASST G. Pini-CTO, Milan, Italy; 3 Fondazione Grigioni per il Morbo di Parkinson, Parkinson, Italy; 4 Gastroenterology/Multivisceral Transplant Unit, Department of Surgery, Oncology, and Gastroenterology, Study Center on Neurodegeneration (CESNE), Padova University, Padova, Italy; 5 Department of general Psychology, Study Center on Neurodegeneration (CESNE), Padova University, Padova, Italy

**Keywords:** Parkinson’s disease (PD), Levodopa-carbidopa intestinal gel (LCIG), Adverse events (AEs), Weight loss (WL), Discontinuation, Mortality

## Abstract

**Introduction:**

Levodopa/carbidopa intestinal gel (LCIG) is an effective treatment in patients with advanced Parkinson’s disease (PD) with consolidated evidence of clinical efficacy. However, only few studies have assessed long-term safety, causes of discontinuation, mortality, and relative predictors.

**Methods:**

We conducted a retrospective analysis of 79 PD patients treated with LCIG between 2005 and 2020 in two Italian Neurological Centers, recording all adverse events (AEs), including weight loss (WL). Kaplan–Meier curve was used to estimate the time to discontinuation and survival. Cox proportional hazard model was employed to identify predictors of discontinuation and mortality, while Pearson’s correlation was used to analyze predictors of WL.

**Results:**

The average follow-up was 47.7 ± 40.5 months and the median survival from disease onset was 25 years. There were three cases of polyradiculoneuropathy Guillain–Barre syndrome-like, all occurred in the early years of LCIG treatment. Twenty-five patients died (32%), 18 on LCIG (including one suicide) and seven after discontinuation. The mean WL was 3.62 ± 7.5 kg, which correlated with levodopa dose at baseline (*p* = 0.002), levodopa equivalent daily dose (LEDD) baseline (*p* = 0.017) and off-duration (*p* = 0.0014), but not dyskinesia. Peristomal complications emerged as a negative predictor of discontinuation (*p* = 0.008).

**Conclusions:**

LCIG has a relatively satisfactory long-term safety profile and efficacy and a relatively low rate of discontinuation. Peristomal complications may represent a predictor of longer duration of therapy. According to the mortality analysis, LCIG patients show a long lifespan. Delaying the initiation of LCIG does not affect the sustainability of LCIG therapy.

**Supplementary Information:**

The online version contains supplementary material available at 10.1007/s00415-022-11269-7.

## Introduction

Levodopa/carbidopa intestinal gel (LCIG) is an effective treatment in advanced Parkinson’s disease (PD) patients, when wearing off, on–off phases, or dyskinesia cause significant functional impairment and make oral medications unable to produce satisfactory motor control [1, 2]. It is widely recognized that changes in peripheral pharmacokinetic parameters, erratic gastric emptying and jejunal absorption, as well as protein competition at intestinal and blood–brain barrier absorption sites contribute to the development of motor complications [3]. LCIG is a combination of levodopa and carbidopa monohydrate (4:1 ratio) in a gel, introduced for the first time in Sweden in 1991, that obtained EU approval in 2004 [4]. Recently, significant efforts have been made to define the eligibility criteria for device-aided therapies and the “5:2:1 rule” (five-times oral levodopa doses/day, 2 h “off” symptoms/day, 1 h of troublesome dyskinesia/day) has been proposed to facilitate patient identification [5, 6]. However, there are no LCIG specific selection criteria or data on long-term safety, additionally identification of predictors of discontinuation and mortality are limited [7–10]. The primary aim of this study was to perform a long-term safety analysis on a large cohort of PD patients treated with LCIG up to 15 years, recording serious adverse events (AEs) and mortality as well as causes and timing of discontinuation. The second aim of this study was to perform a long-term efficacy analysis on motor performance.

## Methods

### LCIG cohort

A retrospective longitudinal study was conducted on 79 patients treated with LCIG between 2005 and 2020 in two Italian tertiary referral neurological centers, specialized in Movement Disorders (Clinica Neurologica of Azienda Ospedaliera di Padova, Padua, and Centro Parkinson e Parkinsonismi, Parkinson Institute, Milan). All medical charts were reviewed in accordance with the prevailing regulations on data protection and privacy. For each subject, the following demographic and clinical data were collected through available medical charts, phone interviews and clinical assessments: current age (for alive patients in LCIG and alive patients who discontinued treatment), gender, ethnicity, age at PD diagnosis, disease duration (calculated as difference between age at PD diagnosis and current age for alive patients; difference between age at PD diagnosis and age at death for deceased patients), age at time of LCIG initiation [patient’s age at time of percutaneous endoscopic gastrojejunostomy (PEG-J) placement for LCIG infusion], LCIG time (time of LCIG exposure), pre-LCIG disease duration (time between age at PD diagnosis and LCIG initiation). We gathered data on naso-jejunal test phase (yes or no), daily duration of LCIG infusion, number of total replacements, previous advanced treatments [apomorphine infusion, Deep Brain Stimulation (DBS)]. PD global cognitive status was assessed through the Mini-Mental Status Examination (MMSE) and Montreal Cognitive Assessment (MOCA) scales.

Moreover, data regarding levodopa dosage, levodopa equivalent daily dose (LEDD) and dopamine agonist equivalent dose (DAED), respectively at baseline or preoperative state, at LCIG initiation, and on follow-up (FU) visits, were obtained. LEDD was calculated by adding to the standard levodopa dose all the other dopaminergic or antiparkinsonian medications converted to the relative potency of standard levodopa [11].

### Long-term safety analysis

All AEs occurred during LCIG treatment were retrospectively reviewed and relative frequency of each event was recorded. We categorized AEs in four groups: peristomal complications (erythema, granulomas, infections, leakage), tube complications (occlusions, dislocations, accidental removal, tube deterioration), other complications [peritonitis, perforation, gut occlusion, weight loss (WL)] and unpredictable events, unrelated to procedure (i.e. cancers, cerebrovascular accidents, cardiac ischemic events). For this purpose we used different sources of information (database, clinical charts, phone interview to family members), investigating the occurrence of relevant events occurred during LCIG exposure. For WL we evaluated baseline body mass index (BMI) and the extent of WL; moreover we investigated potential correlations with the following clinical characteristics: age at LCIG initiation, disease duration (years), LEDD at baseline and at LCIG initiation, levodopa at baseline and LCIG initiation, motor severity according to Hoehn and Yahr (H&Y) stage and Unified Parkinson’s Disease Rating Scale Part III (UPDRS-III), motor fluctuations (UPDRS-IV), BMI, MMSE and MOCA at baseline.

### Discontinuation and mortality investigation

The percentage of patients who discontinued LCIG treatment and the causes of withdrawal were annotated. Temporary discontinuations of LCIG treatment, when rapidly resumed (within 3 months), were not considered. The rate of discontinuation was determined, and multiple baseline variables evaluated as potential predictors of discontinuation: gender, age at PD diagnosis, age at start of LCIG infusion, disease duration at start of LCIG, LEDD, UPDRS-III, H&Y stage, motor fluctuations (UPDRS-IV), Activity of Daily Living (ADL), Instrumental Activity of Daily Living (IADL), BMI, naso-jejunal test phase, MMSE, MOCA, previous advanced therapies (DBS and/or apomorphine infusion), device complications (tube and peristomal complications) and replacements. To capture the oldest population on LCIG treatment, the predictor variable age at LCIG initiation was dichotomized, using a cut-off of 72 years, while for age at PD diagnosis we used a cut-off of 58 years, that represented the third quartile of distribution. We also investigated predictors of mortality, examining the same variables above. Variables with large numbers of missing values (> 50%) were not analyzed.

### Long-term efficacy analysis

In order to assess long-term efficacy, scores obtained at baseline from motor scales (UPDRS-III performed in the ON state and Part IV, H&Y) and UPDRS-II (activities of daily life) were compared to scores at FU visits. Changes in global cognitive functions (MMSE and MOCA) at baseline and at latest FU visit were also compared. We also assessed variation of LEDD and DAED between baseline and at latest FU visit to investigate the presence of any change.

### Statistical methods

Patient demographics at baseline were summarized using descriptive statistics. Quantitative data were expressed as mean ± standard deviation (SD) or median (range), whereas categorical variables were presented as counts and proportions (%). No attempts were made to impute missing values. Kaplan–Meier curve was used to estimate the time to discontinuation of LCIG treatment and the overall median duration of treatment (from LCIG implantation). Similarly, Kaplan–Meier was employed to assess median survival and cumulative percentage of survival in our LCIG population (calculated from PD onset). This analysis accounted for censored observations. Fisher’s exact test was used to compare mortality in two samples of LCIG patients (“patients died on LCIG” vs “patients died some years after discontinuation”). To identify predictors of the time to discontinuation of LCIG treatment and predictors of mortality, we used Cox proportional hazard models. Outcomes of the Cox regression analyses were reported as hazard ratios (HR) with corresponding 95% confidence intervals (95% CI). Subsequently, a multivariate Cox regression analysis was performed including all potential variables having clinical relevance in univariate model (*p* value < 0.05). As WL parameter we calculate WL extent, indicated as difference between the weight recorded at the time of LCIG initiation or within the previous six months (T0) and the weight recorded at time point T1 (subsequent outpatient FU visit after baseline). Pearson’s correlation analysis was used to assess the presence of association between WL extent and clinical baseline characteristics (see above). To assess efficacy, we stratified patients according to FU periods (1st, 2nd, 3rd years of FU and above 4th years of FU) and compared the median of motor and non-motor assessments, LEDD and DAED at FU, with median at baseline records. Comparison of delta changes for each variable at different FU extensions was performed. Data were analyzed using RStudio software (https://www.rstudio.com/).

## Results

### Demographic and clinical features at baseline

A total of 79 advanced PD patients (36 patients from Padua and 43 patients from Milan) received LCIG from 2005 to 2020. All demographic and clinical features are reported in Table 1.

**Table 1:** Most relevant baseline characteristics of LCIG population (mean ± SD)

Baseline clinical features	*N* = 79
Sex (males/females)	47/32
Age at diagnosis (years)	52.7 ± 9.5
Ethnicity	78 Caucasian/1 Hispanic
PD duration (years)	17.8 ± 5.8
Age at start of LCIG infusion (years)	65.6 ± 9.3
Pre-LCIG disease duration (years)	12.9 ± 4.6
Levodopa Baseline (mg)	886 ± 328.7
LEDD baseline (mg)	1333.9 ± 446.4
DAED baseline (mg)	143.9 ± 97
MMSE	26.23 ± 3.4
MOCA	21.4 ± 4.8
UPDRS II	15.4 ± 7.6
UPDRS III	27.2 ± 13.9
UPDRS IV	8.85 ± 3.94
H&Y	2.8 ± 0.9
Naso-jejunal test phase (yes/no)	49/30
ADL	5.21 ± 1.38
IADL	4.4 ± 1.98
Previous advanced therapy	18 (22.8%)
DBS	3 (3.8%)
Apomorphine infusion	15 (19%)

*PD* Parkinson’s disease; *LEDD* levodopa equivalent daily dose, *DAED* dopamine agonist equivalent dose, *MMSE* Mini-Mental Status Examination (corrected score), *MOCA* Montreal Cognitive Assessment (corrected score), *UPDRS* Unified Parkinson’s Disease Rating Scale, *H&Y* Hoehn and Yahr, *ADL* activity of daily living, *IADL* instrumental activity of daily living, *DBS* Deep Brain Stimulation.

### Long-term severe adverse events

We retrospectively recorded all AEs in a subcohort of 63 patients with available data (Table 2). At a mean FU of 47.7 ± 40.5 months (1–173) we found a total of 108 AEs. Most patients reported more than one AE. Peristomal complications occurred in 34 (53.9%), while tube complications were registered in 21 (33.3%). Among peristomal complications, erythema was reported in 22 (34.9%), at least once during LCIG exposure, and 20 patients (31.7%) developed granuloma. Among tube complications, occlusions happened in seven patients (11.1%); dislocations in nine patients (14.3%), accidental removal in two patients (3.2%), tube deterioration in 12 patients (19%) and pump malfunctioning in two patients (3.2%). We recorded one case of gastroenteritis (1.6%), one case of peritonitis (1.6%) and two cases of acute abdomen due to volvulus (3.2%), all requiring hospitalization. Interestingly, during the first years of LCIG implementation, we had three cases of Guillain-Barré syndrome (GBS)-like polyradiculoneuropathy (4.8%), all requiring prompt discontinuation of LCIG with improvement in two cases and one death. There was troublesome dyskinesia in one patient (1.6%), two cases of severe de novo psychosis (3.2%) and one case of difficult to manage impulse disorder (ICD). One patient committed suicide, and another manifested suicidal ideation and was promptly hospitalized. Additional data are reported in Supplementary Fig. 1. AEs led to discontinuations in eight patients (10,12%), while 11 patients (13.9%) discontinued for other reasons. In most cases, AEs causing discontinuations occurred during the first two years after PEG-j implantation. Solid malignant tumors represented one of the most life-threatening events (lung cancer and bladder cancer, respectively diagnosed in two patients), while cerebrovascular and cardiac ischemic events were also recorded. Among 68 patients with available data, the total number of PEG-J replacements was 130, while the overall mean number of procedures for patient was 2.03 ± 2.23 (0–10).

**Table 2:** Long-term AEs in a subcohort of 63 patients presented as numbers and percentage (1st and 2nd column of the table); in the 3rd column n° of patients who discontinued LCIG treatment, due to AEs, is reported; time of discontinuation in months is reported in parenthesis

Long term adverse events (AES)	*N* = 63 Patients	Discontinued
*Peristomal complications*	**34**	
Erythema	22	
Granulation tissue	20	
Peristomal infections	2	2 (24; 81 mo)
Leakage	8	
*Tube complications*	**21**	
Occlusions	8	
Deterioration	10	
Dislocations/Accidental removal	12	
Tube Damage	12	
*Other complication***s**	**13**	
Acute polyradiculoneuropathy	3	3 (5;12; 9 mo)
Intestinal obstructions/volvulus	2	
Gastroenteritis	1	
Peritonitis	1	1 (25 mo)
Duodenal phytobezoar	0	
Septic Shock	2	
Increased dyskinesia	1	1 (20 mo)
ICD	1	
Psychosis	2	1 (6 mo)
Weight loss	26	
*Unpredictable events*	**14**	
Suicide	1	
Ischemic stroke	3	
Atrial fibrillation	1	
Myocardial infarction	1	
Pulmonary embolism	1	
Solid malignancy	2	
DM2/IGT	3	
*COVID-19 infections*	**2**	

*ICD* Impulse Compulsive Disorders (ICD), *DM2* Diabetes Mellitus type 2, *IGT* Impaired Glucose Tolerance

### Weight loss analysis

Weight recording was available in 46 patients, and we observed that PD patients may experience WL during LCIG [12, 13]. The mean WL was 3.62 ± 7.5 kg in a mean LCIG time of 2.1 ± 1.8 years. We registered WL in 26/46 patients (56.52%), comprising a WL > 20 kg in 4 patients, a WL between 10 and 20 kg in 3 patients, between 5 and 10 kg in 5 patients and less than 5 kg in 14 patients. Mean baseline BMI was 23.2 ± 4.8 at T0, while mean BMI at T1 was 20.1 ± 7.2. The number of patients with a BMI < 22 was 17 at baseline and increased to 25 at last observation. Univariate analysis showed a significant correlation with Levodopa baseline dose (*R* = 0.468; *p* = 0.002), LEDD baseline (*R* 0.4101; *p* = 0.017) and off-duration (UPDRS-IV, sub-item 4.3) (*R* = 0.5734; *p* = 0.0014) (Supplementary Fig. 2; Supplementary Table 1). Instead, no correlation was found with other variables examined: age at LCIG initiation, PD duration, levodopa and LEDD at LCIG initiation and FU, UPDRS-III, H&Y stage, MMSE and MOCA (Supplementary Table 1). We found a trend for significance between higher baseline BMI and WL (*R* = − 0.254; *p* value 0.0876) (Supplementary Table 1). However, to clarify if baseline BMI may represent a predictor of weight loss, we performed a cox univariate model using LCIG exposure in months which excluded a significant correlation (HR 1.007, CI 0.799–1.269; *p* value 0.951).

### LCIG discontinuation

During the overall observation period, 19 patients (24.1%) discontinued LCIG infusion, while 25 patients died (31.6%), of which 18/25 on LCIG (72.0%) and 7 some years after discontinuation (28.0%). The causes of discontinuation are listed in Supplementary Table 2. Kaplan–Meier analysis was performed excluding patients deceased on LCIG treatment at the time of death. Discontinuation occurred most commonly in the first 24 months after treatment initiation (Supplementary Fig. 3). Reasons for discontinuation were categorized as follows: device-related, LCIG-related, poor perceived efficacy, poor tolerance, switch to other advanced therapies (apomorphine or DBS) and other unclassified causes. Two patients discontinued due to device-related complications (peristomal infections) and one for peritonitis of unclear origin. Three patients interrupted treatment and opted for DBS. Three patients complaining of poor tolerance refused infusion therapy after a short time, while in two cases treatment was discontinued because of patients’ unsatisfaction and/or subjective inefficacy. Increased dyskinesia was listed as the cause of withdrawal in one patient. Difficulty in managing the infusion system was the cause of discontinuation in another case. According to clinical charts, three patients discontinued infusion temporarily: one for gastroenteritis, promptly resolved; one patient self-discontinued treatment several times for worsening of migraine, not strictly infusion related; one patient for unclear reasons (Supplementary Table 2). Table 3 shows the results of the Cox hazard modeling for the PD group. Initial univariate development of the model revealed that peristomal complications may be a predictor of discontinuation, with a HR of 0.0623 (95% CI 0.0079–0.488; *p* = 0.008). From univariate model, replacements have also emerged as a predictor factor while no additional baseline characteristics was associated with time to discontinuation. Other potential variables were not analyzed due to high number of missing values. At multivariate analysis, replacement had HR 0.5238 (*p* value 0.0511), while peristomal complications had HR 0.0696 (*p* = 0.0117), confirming its role as potential predictor of discontinuation. To evaluate the impact of peristomal complications on discontinuation we analyzed each type of peristomal complication separately using the long-rank test to compare the discontinuation time respectively between the group with erythema and without erythema, granuloma and without granuloma, leakage and without leakage (Supplementary Fig. 4). From long-rank test, erythema and granuloma emerged as negatively associated with discontinuation, while leakage did not reach statistical significance. Median of discontinuation for population with occurrence of erythema was 144 months (81-NA), while for the population without erythema was 62 (52-NA); a log-rank test of the difference between the two curves (erythema “yes” and erythema “no”) yields *χ*^2^ = 10,7; *p* value = 0.001. Median of discontinuation for population who presented history of granuloma was 144 (144-NA), while from the population without granuloma was 65 (52–131); the log-rank test (granuloma “yes” and granuloma “no”) yields *χ*^2^ = 4.1; *p* value = 0.04. Median of discontinuation for population with history of leakage was 151 months (60-NA), for the counterpart was 81 months (62–144); the log-rank test (leakage “yes” and leakage “no”) yields *χ*^2^ = 1.8; *p* value = 0.2 (Supplementary Fig. 4).

**Table 3:** Cox univariate analysis for predictors of discontinuation and multivariate analysis for statistically significant (p < 0.05) variables

Variable	Univariate model			Multivariate model		
	HR	CI	*p* value	HR	CI	*p* value
Sex (male vs female)	0.649	(0.263–1.602)	0.349			
*Age at PD diagnosis* (≥ 58 years vs < 58 years)	1.326	(0.468–3.375)	0.596			
PD duration (yrs.)	1.037	(0.956–1.124)	0.377			
Nasojejunal test phase	1.075	(0.4066–2.843)	0.884			
Prior therapy	0.525	(0.161–1.719)	0.287			
Replacements	0.343	(0.167–0.704)	**0.0035**	0.5238	(0.2734–1.003)	0.0511
Levodopa baseline	0.999	(0.998–1.001)	0.43			
DEAD baseline	0.997	(0.982–1.013)	0.718			
LEDD baseline	0.999	(0.998–1.002)	0.651			
Age at LCIG initiation (≥ 72 years vs < 72 years)	1.136	(0.358 -3.598)	0.828			
BMI	1.007	(0.799–1.269)	0.95			
Peristomal complications (yes vs no)	0.0623	(0.0079–0.488)	**0.008**	0.0696	(0.0087–0.5531)	**0.0117**
Tube complications (yes vs no)	0.332	(0.072–1.523)	0.156			
MMSE	1.02	(0.877–1.186)	0.792			
MOCA	0.895	(0.746–1.072)	0.229			
H&Y baseline	0.839	(0.0.468–1.505)	0.556			
ADL	2.684	(0.349–20.62)	0.342			
IADL	1.574	(0.837–2.959)	0.159			
UPDRS-III	0.962	(0.918–1.007)	0.094			

Bold values denote statistical significance at the *p* < 0.05

*PD* Parkinson’s disease, *yrs.* Years, *DEAD* dopamine agonist equivalent dose, *LEDD* levodopa equivalent daily dose, *LCIG* levodopa/carbidopa intestinal gel, *BMI* body mass index, *MMSE* Mini-*Mental Status* Examination, *MOCA Montreal Cognitive Assessment*, *H&Y* Hoehn and Yahr, *ADL* activities of daily living, *IADL* Instrumental activities of daily living, *UPDRS* Unified Parkinson’s Disease Rating Scale

### LCIG mortality

**Fig. 1:**
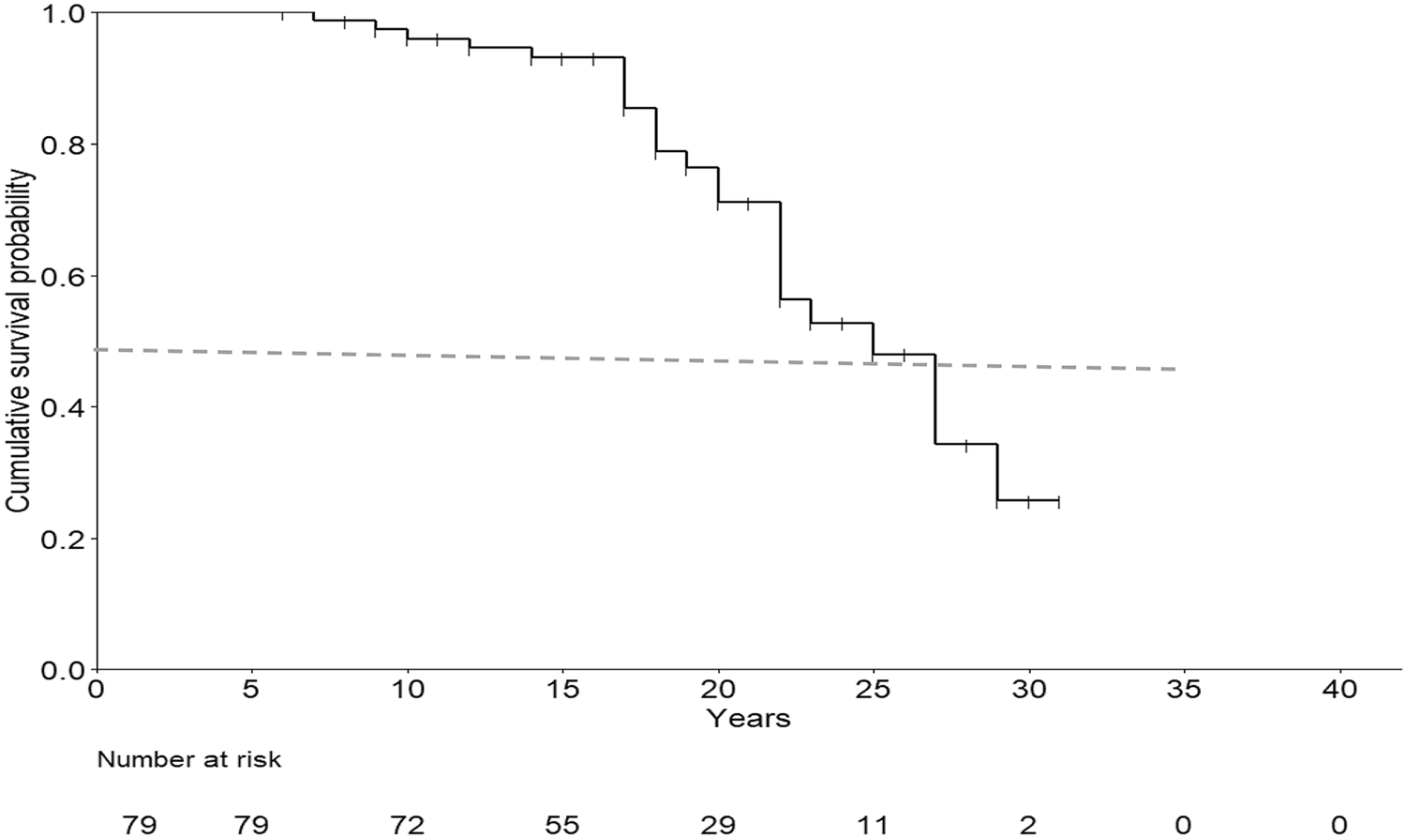
Kaplan–Meier curve showing cumulative survival probability in LCIG patients. The vertical tick marks denote censored observations. A median time of survival from PD onset of 25 years, (CI 95%: 22-na) is showed

Among 79 patients, the event “death” was recorded in 25 patients (31.6%). Kaplan–Meier survival analysis indicated a median time of survival from the age of PD onset of 25 years (CI 95%: 22-na). The cumulative probability of survival was 100% within six years of disease, slightly decreasing to 98% after seven years and below 80% only after 17 years of disease duration, suggesting a high probability of survival in our LCIG population (Fig. 1). Overall, the mean age at death was 73.1 years. The mean age at death for patients who died on LCIG therapy (*n* = 18) was 73.36 ± 8.05 years, while for those who died after LCIG discontinuation (*n* = 7) was 72.833 ± 12.45 years. Age at death did not differ between the two subgroups (*p* = 0.54).

Reasons of death were considered mostly unrelated to device/LCIG therapy (Supplementary table 3). Analysis on predictors of mortality showed that older age at PD diagnosis (HR 4.554; 95% CI 1.796–11.55; *p* = 0.001), older age at LCIG initiation (HR 3.198; 95% CI 1.337–7.651; *p* = 0.009), pre-LCIG disease duration (HR 0.838; 95% CI 0.753–0.934; *p* = 0.001) and naso-jejunal test phase (HR 4.742; CI 11–20.25; *p* = 0.04) are associated with a greater mortality risk. No association was found for sex and the other variables examined. The multivariate model confirmed pre-LCIG disease duration as a negative predictor of mortality (HR 0.393; 95% CI 0.164–0.940; *p* = 0.036), while all the others predictor variables analyzed did not reached statistical significance (Supplementary Table 4).

### Long-term efficacy

Data of 45 patients were analyzed through stratification in five sub-groups, according to FU length (Supplementary Fig. 5). Comparison of delta changes for each variable at different FU extensions (supplementary Table 5) showed a global reduction of DAED and an increase of levodopa dose (reflecting higher doses of daily LCIG), while LEDD remained stable along the disease course. We also detected a maintenance of global cognitive performance. An improvement of UPDRS-III around the 3rd–4th year of FU was also noticed. Above the 4th year a worsening of (UPDRS-III) and a worsening of UPDRS-I and II scores were detected, consistent with the progression of the disease, while H&Y remained stable along disease course (Supplementary Fig. 6).

## Discussion

We analyzed an Italian cohort of advanced PD patients treated with LCIG to enrich our knowledge regarding long-term effects of this treatment. Although LCIG efficacy is well recognized, studies assessing its long-term safety profile, discontinuation and mortality are lacking. We detected few AEs, mostly peristomal complications such as erythema and granulation tissue, and a very low prevalence of peristomal infections, similar to previous studies [4, 14, 15]. We also recorded three cases of GBS-like polyradiculoneuritis, all occurred in the early years of LCIG therapy, before our and other research groups reported this complication and suggested vitamin B complex supplementation [8]. In our cohort, AEs led to discontinuation in eight patients (10,12%); these included worsening of dyskinesia and ICD. Peristomal infections led to discontinuation in two cases as well as peritonitis in one patient. We acknowledge that the number of AEs recorded may be underestimated due to retrospective nature of the study and the heterogeneity of data collection. However, since our health authorities require recording all adverse events in a dedicated patient registry, it is likely that we captured the most significant events. We found a relatively low rate of discontinuation (24%) which occurred mostly in the first two years after starting LCIG infusion, in line with previous studies [10, 16]. Differently from previous studies [4, 10], we did not consider death as a reason of withdrawal but analyzed mortality separately. Conversely, if we include death as a reason of discontinuation, we had 37 discontinuations, of which 18 due to death occurred for unpredictable events. We assessed several baseline clinical characteristics as potential predictors of discontinuation. From our data peristomal complication of severe entity, including infections and peritonitis represented a reason of withdrawal. However, mild peristomal complications such as erythema and granuloma emerged as negative predictors of discontinuation and were associated with a long duration of therapy. This complication is common and can be managed if patients are strictly followed by dedicated personnel. Unlike previous studies, disease duration and gender were not predictors of discontinuation [4, 10]. Furthermore, it is important to highlight that age at LCIG initiation was also not associated with a significant risk of discontinuation, reinforcing the concept that LCIG infusion is a suitable therapeutic strategy in elderly patients [17]. Another difference with previous studies [9] concerns the prognostic value of baseline cognitive assessments (MMSE and MOCA), which did not affect either discontinuation or mortality although in our cohort no patient had severe cognitive deficits at time of treatment initiation (mean age at PD onset was 56 years while age at LCIG initiation was 65 years). In a recent retrospective study, Viljaharju et al. found that patients living alone are more prone to discontinuation, especially during the first year of discontinuation. However none of our patients lived alone at LCIG initiation, since in both centers the lack of a caregiver was considered a contraindication. We found an overall discontinuation of 24.1%, which is slightly lower than that found by Viljaharju et al. (27.2%). The number of severe adverse events registered in the long term was similar, with only one death related to LCIG/device [18].

Moreover, safety analysis showed a high recurrence of WL in LCIG patients, similarly to previous studies [12, 13, 19]. Interestingly, WL extent correlated with baseline levodopa and LEDD, and off-duration also emerged as a potential predictor. However, differently from Fabbri et al. [19], no association was observed with the severity of dyskinesia. These findings may imply a possible role of malnutrition and/or malabsorption in PD patients treated with LCIG but we cannot exclude an effect on gut permeability and intestinal microbiota [20, 21]. Our study allows to rule out an effect of the baseline BMI on weight loss, which seems to occur independently by the presence of initial overweight.

Interestingly, analysis of mortality showed that LCIG is associated with long survival, with a median of 25 years from PD onset, which is significantly greater than median survival time reported in literature [22–24].

However, age at death was almost 10 years earlier than the general Italian population (82.4 years in 2020) but did not differ between men and women and was longer that what it was reported in the PRIAMO study [25].

The main reasons of death were mainly pulmonary infections, which are still common in late-stage PD and are likely to be related to disease progression rather than to device or infusion. Moreover, from our findings pre-LCIG duration emerged as a negative predictor of mortality, confirming that an old age is not a limiting factor for LCIG. More studies are needed to corroborate the possible effect of LCIG on survival, especially in the elderly population.

In addition to a satisfactory safety profile, our data confirmed a sustained long-term efficacy with a maintenance of cognitive performance in the long term. We observed a stable LEDD over the years, with a reduction in dopamine-agonists use and a slight increase of levodopa.

We acknowledge that our study has limitations mainly the missing data and the retrospective design. However, we believe it provides useful evidence on the long-term follow-up of patients on LCIG which may help providing a proper indication on prognosis and outcome including caregiver burden and survival [26].

## Conclusions

In conclusion, we determined that LCIG is associated with a relatively satisfactory long-term safety profile and that peristomal infections represented one of the most relevant causes of discontinuation, together with acute peripheral polyradiculoneuritis. However, mild-moderate entity peristomal complications such as erythema and granuloma do not impact on the sustainability of LCIG therapy. In our study, the observation of significant WL, which correlated to levodopa dose and not dyskinesia, may suggest the possibility of malabsorption and malnutrition secondary to levodopa infusion. However, this important AE may also be related to a combination of aging and disease progression and deserves specific studies focused on the implementation of physical activity, nutritional support and regular assessment of weight and nutritional status in these patients. Mortality analysis showed that LCIG patients have a long lifespan, but they still die almost 10 years earlier than their unaffected Italian counterparts. The only study assessing the mortality in LCIG patients found a lifespan of 18 years since PD onset [9], which is lower than the cumulative survival in our LCIG population, but similarly longer than median survival in PD patients [22–24, 27]. Time to initiation of LCIG therapy may vary and patients with earlier onset may be less prone to undergo a PEG procedure and are generally more likely to opt for deep brain stimulation. Further studies should evaluate the impact of device aided therapies on lifespan and assess factors that may be relevant for survival.

## Supplementary Information

Below is the link to the electronic supplementary material.

Supplementary file1 (DOCX 1058 KB)
