# Correction to: Long-term safety, discontinuation and mortality in an Italian cohort with advanced Parkinson’s disease on levodopa/carbidopa intestinal gel infusion

**DOI:** 10.1007/s00415-022-11321-6

**Published:** 2022-08-20

**Authors:** Federica Garrì, Francesco Paolo Russo, Tommaso Carrer, Luca Weis, Francesca Pistonesi, Michele Mainardi, Michele Sandre, Edoardo Savarino, Fabio Farinati, Francesca Del Sorbo, Paola Soliveri, Daniela Calandrella, Roberta Biundo, Miryam Carecchio, Anna Lena Zecchinelli, Gianni Pezzoli, Angelo Antonini

**Affiliations:** 1 Parkinson and Movement Disorders Unit, Study Center on Neurodegeneration (CESNE), Department of Neuroscience, University of Padua, Padova, Italy; 2 Parkinson Institute, ASST G. Pini-CTO, Milan, Italy; 3 Fondazione Grigioni per il Morbo di Parkinson, Milan, Italy; 4 Gastroenterology/Multivisceral Transplant Unit, Department of Surgery, Oncology, and Gastroenterology, Study Center on Neurodegeneration (CESNE), Padova University, Padova, Italy; 5 Department of General Psychology, Study Center on Neurodegeneration (CESNE), Padova University, Padova, Italy

## Correction to: Journal of Neurology 10.1007/s00415-022-11269-7

The original version of this article unfortunately contained a mistake. The affiliation details for following author’s Tommaso Carrer, Luca Weis, Francesca Pistonesi, Michele Mainardi, Michele Sandre, Francesca Del Sorbo, Paola Soliveri, Daniela Calandrella, Miryam Carecchio, Gianni Pezzoli and Angelo Antonini were incorrectly given.

The correct affiliations are indicated below.

Tommaso Carrer: Parkinson and Movement Disorders Unit, Study Center on Neurodegeneration (CESNE), Department of Neuroscience, University of Padua, Padova, Italy.

Luca Weis: Parkinson and Movement Disorders Unit, Study Center on Neurodegeneration (CESNE), Department of Neuroscience, University of Padua, Padova, Italy.

Francesca Pistonesi: Department of General Psychology, Study Center on Neurodegeneration (CESNE), Padova University, Padova, Italy.

Michele Mainardi: Parkinson and Movement Disorders Unit, Study Center on Neurodegeneration (CESNE), Department of Neuroscience, University of Padua, Padova, Italy.

Michele Sandre: Parkinson and Movement Disorders Unit, Study Center on Neurodegeneration (CESNE), Department of Neuroscience, University of Padua, Padova, Italy.

Francesca Del Sorbo: Parkinson Institute, ASST G. Pini-CTO, Milan, Italy.

Paola Soliveri: Parkinson Institute, ASST G. Pini-CTO, Milan, Italy.

Daniela Calandrella: Fondazione Grigioni per il Morbo di Parkinson, Italy.

Miryam Carecchio: Parkinson and Movement Disorders Unit, Study Center on Neurodegeneration (CESNE), Department of Neuroscience, University of Padua, Padova, Italy.

Gianni Pezzoli: Fondazione Grigioni per il Morbo di Parkinson, Italy.

Angelo Antonini: Parkinson and Movement Disorders Unit, Study Center on Neurodegeneration (CESNE), Department of Neuroscience, University of Padua, Padova, Italy.

The original article has been corrected.

