# Supplementary material for: Long-term safety, discontinuation and mortality in an Italian cohort with advanced Parkinson’s disease on levodopa/carbidopa intestinal gel infusion
Source: J Neurol. 2022 Jul 25;269(10):5606–14. doi: 10.1007/s00415-022-11269-7 (PMC9309989; doi:10.1007/s00415-022-11269-7)
Supplement: Supplementary file 1 — Supplementary file1 (DOCX 1058 KB) [file 415_2022_11269_MOESM1_ESM.docx]

**Supplementary Figures**

*Supplementary Fig. 1. Distribution of long-term AEs in a subcohort of 63 patients on LCIG.*


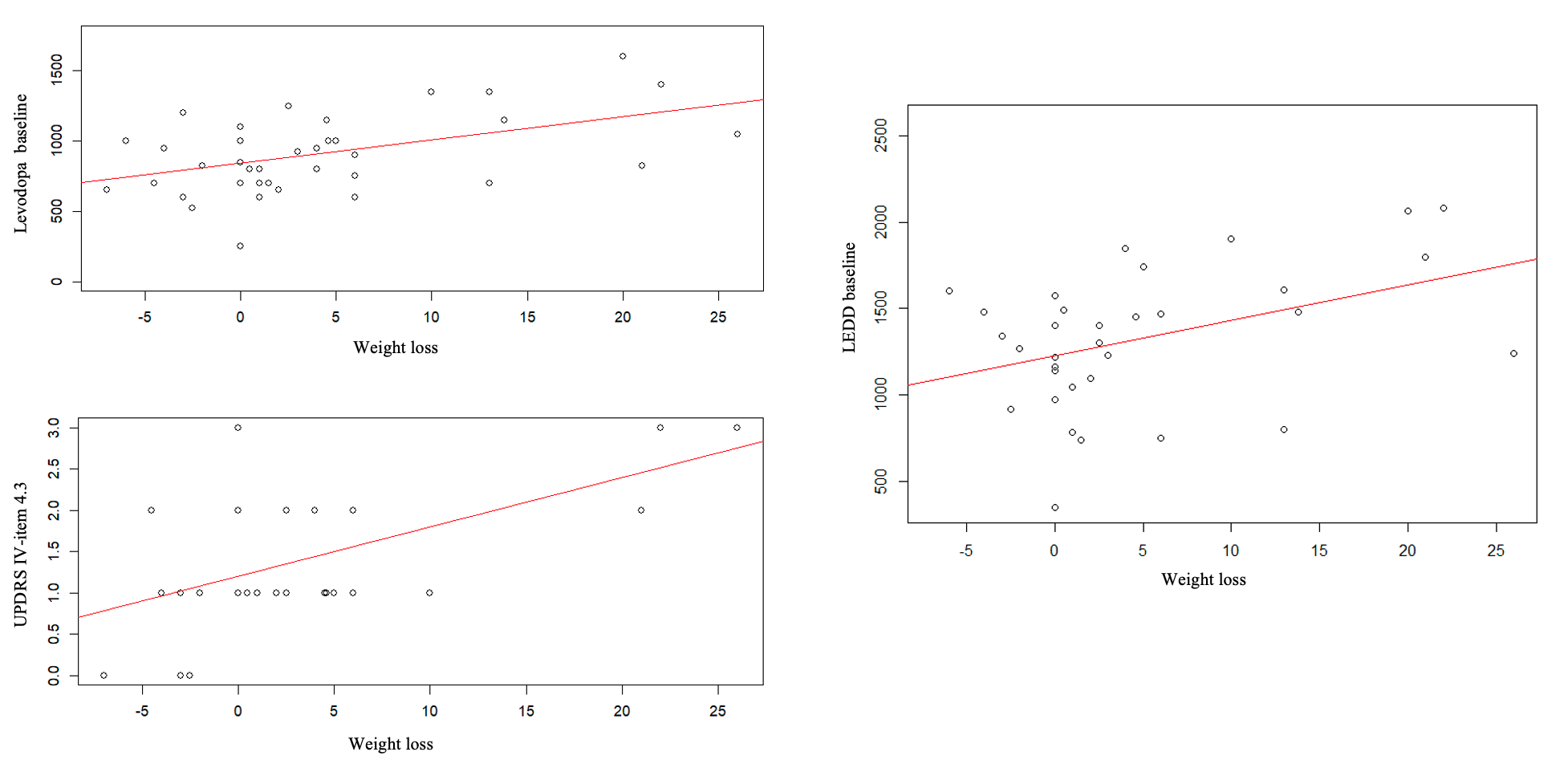


*Supplementary Fig. 2. Pearson correlation analysis graph between WL extent and Levodopa baseline (R=0.468; p=0.002),LEDD baseline (R 0.4101; p=0.017)and UPDRS-item 4.3 and off-duration (UPDRS IV-item 4.3) (R=0.5734; p=0.0014).*


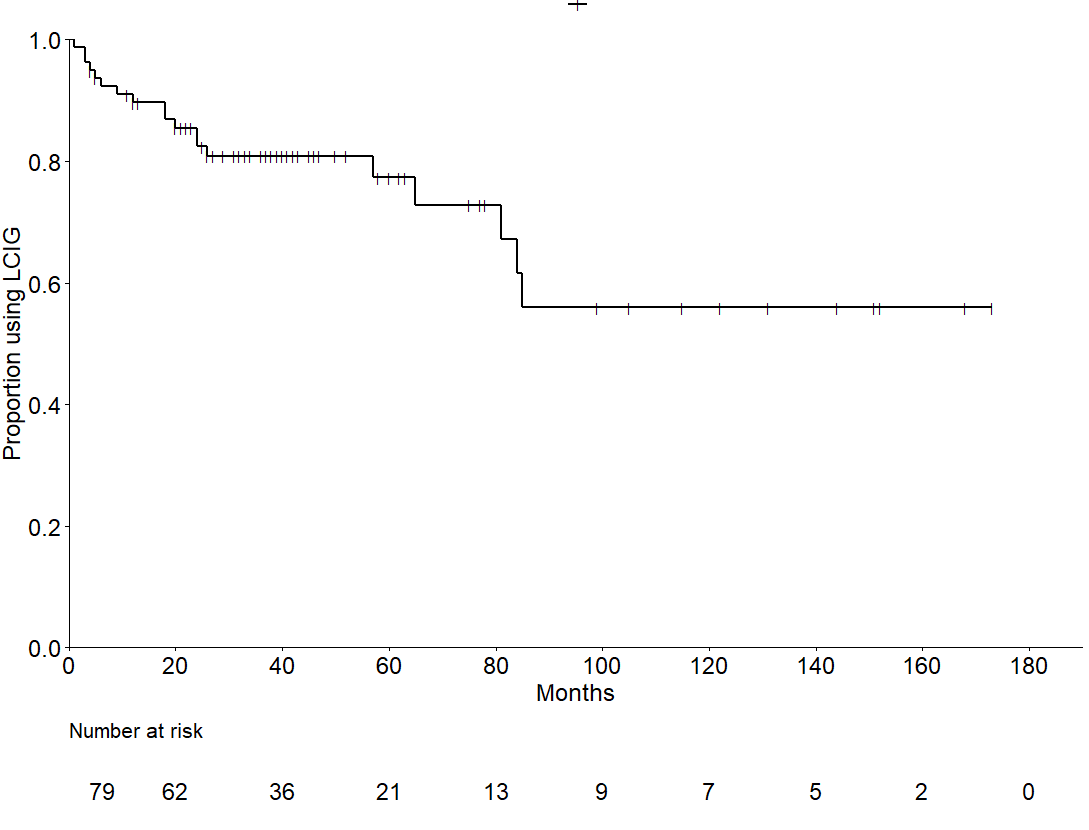


*Supplementary Fig. 3. Kaplan–Meier curve showing time to discontinuation of LCIG infusion. The vertical tick marks denote censored observations.*


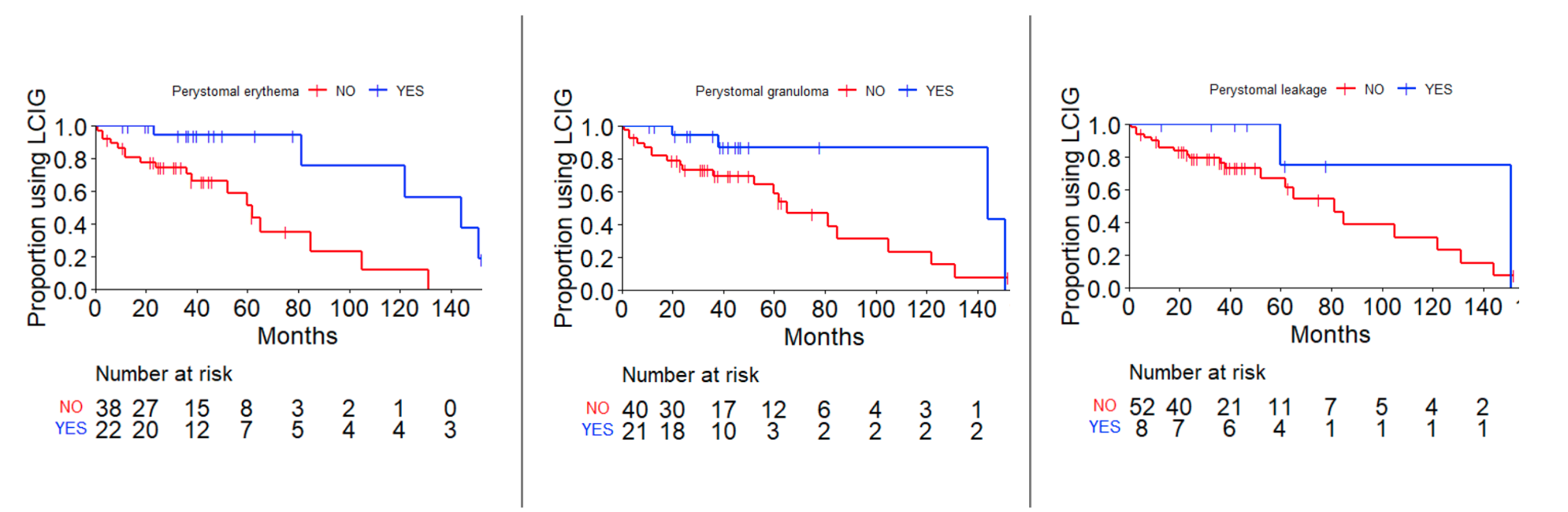


*Supplementary Fig. 4 Analysis of the impact of each type of peristomal complication (erythema, granuloma and leakage) on discontinuation time. (Long rank test).*


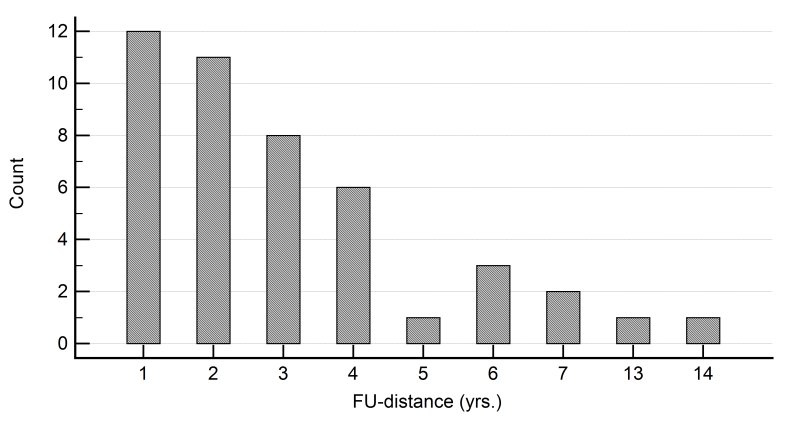


*Supplementary Fig.5 FU length in LCIG patients for N=45 PD patients* *(12 patients (*26.7%) *one year-FU, 11 patients(24.4%) two years-FU, eight patient (17.8%) three years-FU, six patients (13.3%) four years-FU and eight patients (17.8%) more than four years-FU).*


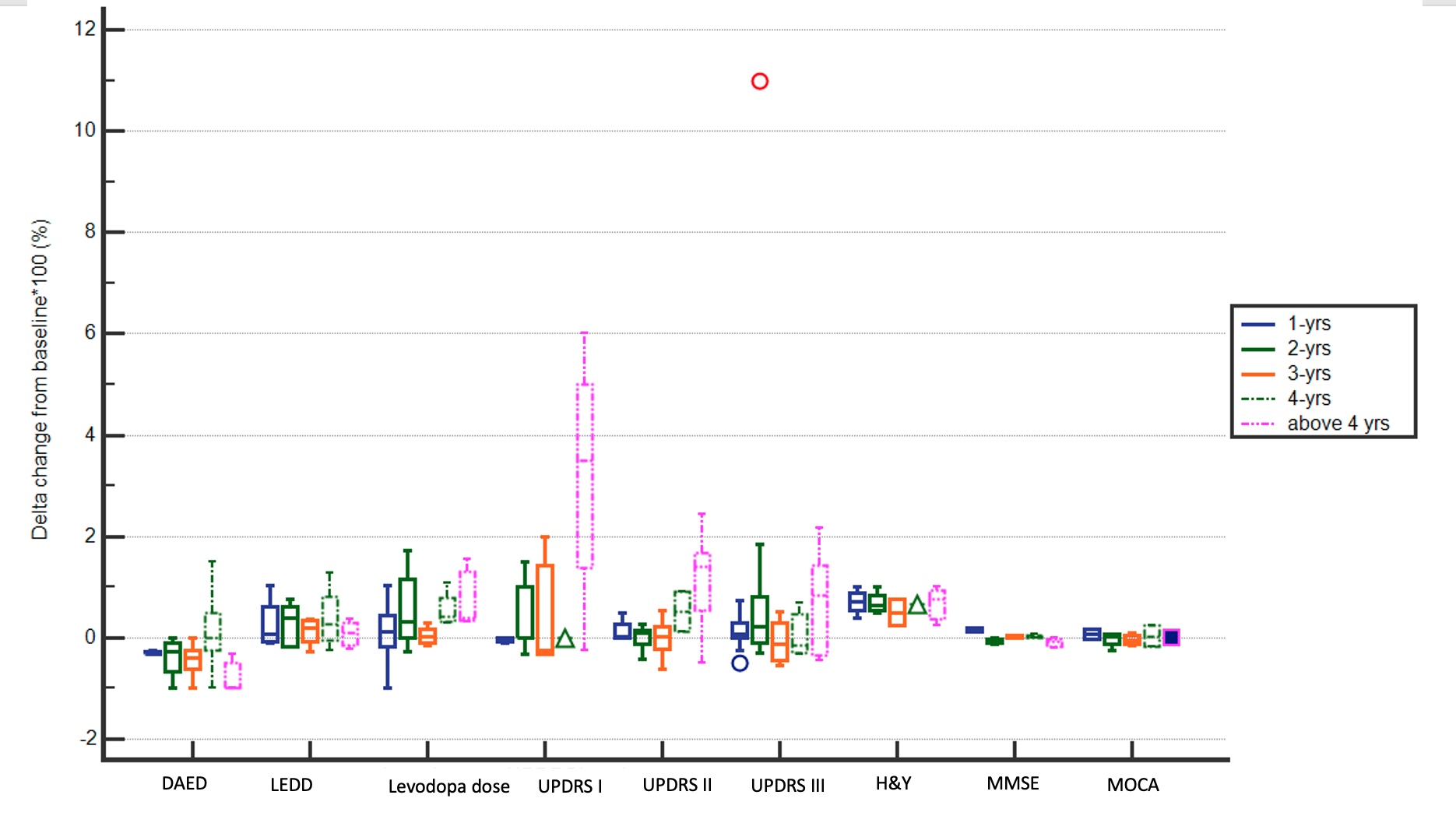


*Supplementary Fig.6. Short-term and long-term delta changes from baseline for drug dosages, motor and non-motor scales (UPDRSI, UPDRSII, UPDRSIII, H&Y) and cognitive scales adjusted for age and education (MMSE, MOCA).*

**Supplementary Tables**

|  | Weight loss |  |
| --- | --- | --- |
| Variable | **R coefficient** | **p-value** |
| PD duration | -0.2092 | 0.163 |
| Age at LCIG initiation | 0.064 | 0.6963 |
| Baseline BMI | -0.254 | 0.0876 |
| LEDD baseline | 0.4101 | **0.017** |
| LEDD LCIG initiation | 0.2366 | 0.185 |
| Levodopa baseline | 0.468 | **0.002** |
| Levodopa LCIG initiation | 0.349 | 0.054 |
| Levodopa LCIG FU | 0.2995 | 0.095 |
| LEDD FU | 0.236 | 0.185 |
| H&Y | 0.0368 | 0.821 |
| UPDRS-III | -0.1031 | 0.532 |
| UPDRS-IV | 0.1278 | 0.663 |
| Item 4.1 baseline | 0.1261 | 0.522 |
| Item 4.2 baseline | 0.1587 | 0.419 |
| Item 4.3 baseline | 0.5734 | **0.0014** |
| MMSE | 0.0592 | 0.728 |
| MOCA | 0.017 | 0.9325 |

*Supplementary Table 1. Pearson’s correlation analysis (univariate model) for all variables listed with WL extent.*

| MAIN REASON OF DISCONTINUATION | N=19 |
| --- | --- |
| Device Related Complications | 2 |
| LCIG Related AEs | 3 |
| Poor Tolerance | 3 |
| Subjective Inefficacy/Unsatisfaction | 2 |
| Switch To Other Therapies (DBS) | 3 |
| Difficulty To Manage Pump | 1 |
| Peritonitis | 1 |
| Increased Dyskinesia | 1 |
| Psychosis | 1 |
| Unknown | 2 |

*Supplementary Table 2. Main reasons of discontinuation of treatment in patients who discontinued LCIG infusion (N=19).*

| Reasons of death | N=18 |
| --- | --- |
| LCIG related | 1 |
| Device related | 0 |
| Unrelated to device/LCIG | 17 |
| *Suicide* | 1 |
| *Comorbidities* | 1 (PE in NHL) |
| *Cardiac arrest* | 1 |
| *Deterioration in PD* | 2 |
| *Pneumonia* | 8 |
| *Septic shock* | 1 |
| *Unspecified* | 3 |

*Supplementary Table 3. Main reasons of death in LCIG population in course of treatment. Abbreviations: PE=* *Pulmonary*embolism; *NHL=*non-Hodgkin lymphoma; PD=Parkinson’s Disease.

| Univariate model | | |  | | | Multivariate model | | |
| --- | --- | --- | --- | --- | --- | --- | --- | --- |
| VARIABLE | HR | CI | | p-value | HR | | CI | p-value |
| Sex (male vs female) | 0.829 | (0.370-1.86) | | 0.3 |  | |  |  |
| Age at PD diagnosis  (≥58 yrs. vs <58 yrs.) | 4.554 | (1.796-11.55) | | **0.001** | 1.617 | | (0.699-3.7366) | 0.261 |
| Disease duration | 2.113 | (0.929-4.804) | | 0.07 |  | |  |  |
| Naso-jejunal test phase (yes vs no) | 4.742 | (11-20.25) | | **0.04** | 4.124 | | (0.951-17.894) | 0.058 |
| Replacements | 1.147 | (0.963-1.366) | | 0.1 |  | |  |  |
| Levodopa baseline | 0.999 | (0.997-1.001) | | 0.3 |  | |  |  |
| DEAD baseline | 0.996 | (0.986-1.006) | | 0.4 |  | |  |  |
| LEDD baseline | 0.999 | (0.997-1.001) | | 0.6 |  | |  |  |
| Age at LCIG initiation  (≥72 yrs. vs <72 yrs.) | 3.198 | (1.337-7.651) | | **0.009** | * | | * | * |
| Pre-LCIG disease duration  (≥13 yrs.) | 0,838 | (0,753-0,934) | | 0,001 | 0.393 | | (0.264-0.940) | **0.036** |
| BMI | 0.859 | (0.696-1.06) | | 0.2 |  | |  |  |
| Peristomal complications  (yes vs no) | 0.568 | (0.201-1.604) | | 0.3 |  | |  |  |
| H&Y baseline | 0.983 | (0.631-1.53) | | 0.9 |  | |  |  |
| MMSE | 0.975 | (0.872-1.09) | | 0.657 |  | |  |  |
| MOCA | 1.069 | (0.831-1.374) | | 0.6 |  | |  |  |
| ADL | 1.377 | (0.509-3.724) | | 0.528 |  | |  |  |
| IADL | 0.933 | (0.548-1.588) | | 0.8 |  | |  |  |
| UPDRS-III | 1,007 | (0,977-1,038) | | 0,6 |  | |  |  |
| UPDRS-IV | 1,056 | (0,746-1,495) | | 0,758 |  | |  |  |

*Supplementary Table 4. Association between baseline clinical characteristics and mortality using Cox regression univariate model and multivariate analysis for statistically significant (*p*< 0.05) variables. Abbreviations: PD=Parkinson’s disease; yrs.=years; DEAD=*dopamine agonist equivalent dose*; LEDD=* levodopa equivalent daily dose*; LCIG=levodopa/carbidopa intestinal gel; BMI=body mass index; MMSE=* Mini-Mental Status Examination*; MOCA=*Montreal Cognitive Assessment*; H&Y=* Hoehn and Yahr*; ADL=Activities of Daily Living; IADL=Instrumental Activities of Daily Living; UPDRS=* Unified Parkinson’s Disease Rating Scale. * Age at LCIG initiation was excluded in the multivariate analysis since there was a lack of  convergence if all the variable were included due to poorly fitting observations.


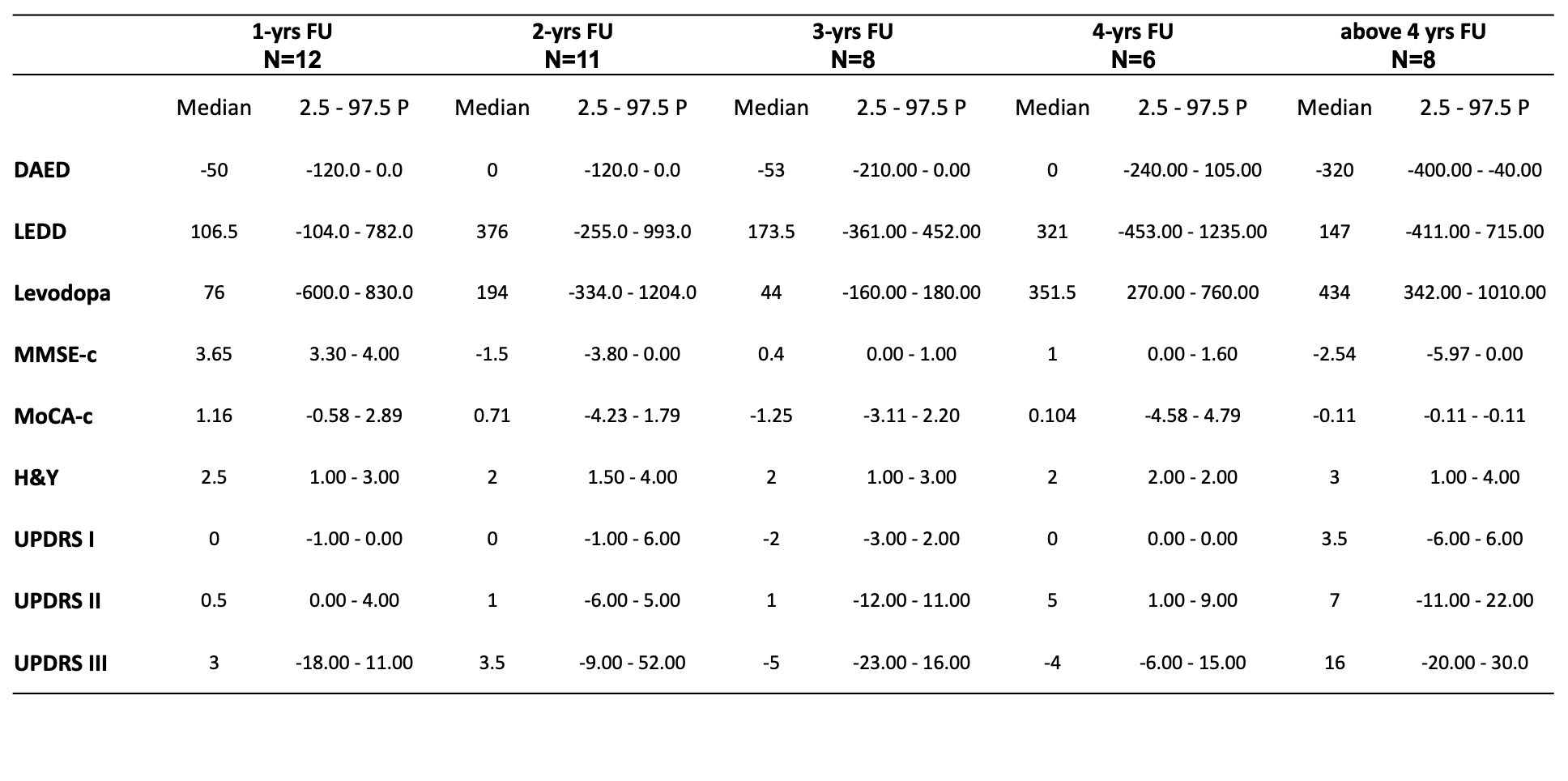
*Supplementary Table. 5. Short-term and long-term delta changes from baseline for drug dosages, motor and non-motor scales (UPDRS I, UPDRS II, UPDRS III, H&Y) and cognitive scales adjusted for age and education (MMSE, MOCA) in accordance with FU extension.*
